# Targeted Therapy for Melanomas Without BRAF V600 Mutations

**DOI:** 10.1007/s11864-022-00946-4

**Published:** 2022-04-05

**Authors:** Christian Menzer, Jessica C. Hassel

**Affiliations:** Section of DermatoOncology, Department of Dermatology and National Center for Tumor Diseases (NCT), University Hospital Heidelberg, Im Neuenheimer Feld 460, 69120 Heidelberg, Germany

**Keywords:** Melanoma, MAPK pathway, BRAF, MEK, Targeted therapy, BRAF mutation, V600, Non-V600, Trametinib, Binimetinib, Cobimetinib, C-kit, NRAS, Imatinib

## Abstract

Modern therapy of advanced melanoma offers effective targeted therapeutic options in the form of BRAF plus MEK inhibition for patients with BRAF V600 mutations. For patients lacking these mutations, checkpoint inhibition remains the only first-line choice for treatment of metastatic disease. However, approximately half of patients do not respond to immunotherapy, requiring effective options for a second-line treatment. Advances in genetic profiling have found other possible target molecules, especially a wide array of rare non-V600 BRAF mutations which may respond to available targeted therapy.

More information on the characteristics of such mutants is needed to further assess the efficacy of targeted therapies in the metastatic and adjuvant setting of advanced melanoma. Thus, it may be helpful to classify known BRAF mutations by their kinase activation status and dependence on alternative signaling pathways. While BRAF V600 mutations appear to have an overall more prominent role of kinase activity for tumor growth, non-V600 BRAF mutations show great differences in kinase activation and, hence, response to BRAF plus MEK inhibition. When BRAF-mutated melanomas rely on additional signaling molecules such as RAS for tumor growth, greater benefit may be expected from MEK inhibition than BRAF inhibition. In other cases, mutations of c-kit or NRAS may serve as important pharmacological targets in advanced melanoma. However, since benefit from currently available targeted therapies for non-V600 mutants is usually inferior regarding response and long-term outcome, checkpoint inhibitors remain the standard recommended first-line therapy for these patients.

Herein, we review the current clinical data for characteristics and response to targeted therapy of melanomas lacking a V600 BRAF mutation.

## Introduction

The detection of the MAP kinase/ERK signaling pathway has, aside from the discovery of immune checkpoint inhibitors, revolutionized the principles of modern therapy in advanced melanoma. Tumor proliferation in up to half of all melanomas relies on activating BRAF mutations [1, 2], of which 70–80% account for mutations in V600E and another 10–20% for the less active V600K [3••, 4–8]. Prolonged progression-free survival (PFS) and overall survival (OS) with BRAF inhibitors plus MEK inhibitors was shown for all approval studies. Yet, these studies usually only included BRAF V600E and V600K [Combi-D (dabrafenib/trametinib) [4••] and Columbus (encorafenib/binimetinib) [3••] or only BRAF V600E [CoBRIM (vemurafenib/cobimetinib) [9••] mutated melanomas.

Broader accessibility to next-generation sequencing in advanced melanoma revealed further BRAF mutations in other V600 or non-V600 gene locations [10]. Rare activating mutations account for approximately 3.4 to 14% of BRAF-mutated melanomas [2, 5•, 8, 11–12]. Due to the low volume of patients and their exclusion from large drug approval studies, there is a lack of data regarding response and survival benefit of these mutations to inhibition of the MAPK pathway. However, currently available data suggest a less favorable course of disease in patients with BRAF mutations other than V600E and V600K, especially in patients harboring a non-V600 mutation [13••].

Advances in the genetic assessment of tumor-driving oncogenes allow for a categorization of BRAF mutants into three classes based upon their kinase activity, ability to signal as monomers or dimers, and dependency on RAS signaling [14••]. Other frequent oncogenic mutations in metastatic melanoma, which may serve as a therapeutic target, include RAS, NF1, or KIT [2].

Further research is warranted to improve the therapeutic arsenal for these mutations and find additional targets that may help control melanoma cells in the future.

In this review, current treatment options with targeted therapy for patients without V600 BRAF mutations will be discussed. This overview aims to facilitate clinicians’ treatment decisions in melanoma patients who are not eligible for therapy with checkpoint inhibitors.

## Rare BRAF mutations

Approximately 50% of melanomas are driven by an activating BRAF mutation which serves as an essential kinase in the mitogen-activated protein kinase (MAPK) pathway responsible for tumor cell proliferation [1]. Of these BRAF-mutated melanomas, up to 80% carry a mutation in the V600E and another 10–20% in the V600K gene [6]. Prolongation of PFS and OS after 5 years has been shown for 19% and 34% of melanoma patients with BRAF V600E and V600K mutations when treated with dabrafenib plus trametinib (COMBI-d and COMBI-v) [15•] and for 14% and 31% of patients with V600E-mutated melanoma treated with cobimetinib plus vemurafenib (coBRIM) [16•], respectively. Hence, on par with immune checkpoint inhibitors, these are the recommended first-line therapies for advanced melanomas harboring a BRAF V600E or V600K mutation.

Due to their rare nature and exclusion from larger drug approval studies, fewer data exist on the efficacy of BRAF inhibition and/or MEK inhibition in less frequent BRAF mutants, which account for approximately 3.4–14% of BRAF-mutated melanomas [2, 5•, 8, 11–12]. Data for unique BRAF mutants may be obtained from case reports or case series of patients with advanced melanomas, as well as other tumor entities for which BRAF mutations have been described, such as thyroid, colorectal, or non-small cell lung cancer (NSCLC) [14••].

In normal cells, the mitogen-activated protein kinase (MAPK) pathway is initiated by ligand-mediated activation of receptor tyrosine kinases (RTKs), which subsequently induce RAS GTPases (NRAS, KRAS, HRAS), followed by activation through dimerization of RAF proteins (ARAF, BRAF, CRAF) [14••]. Downstream activation of MEK1/2 and then ERK1/2 by phosphorylation stimulates transcription factors responsible for cell proliferation and survival. Mutation in one of the components of the MAPK pathway may result in uncontrolled cell proliferation and subsequent tumor growth.

Historically, classification of BRAF mutations merely referred to their degree of kinase activation [14••]. Recently, the characterization of BRAF mutants has become more sophisticated, considering differences in kinase activity, RAS dependency, and activation through dimerization. Thus, a three-class system was developed with class I containing mostly mutants signaling as monomers, class II relying on activation from dimers, and class III consisting of BRAF mutations with kinase-inactivating heterodimers [17•–18••].

BRAF mutation class I contains the most common structural BRAF mutations with high BRAF and downstream MAPK pathway activation [14••]. V600 mutations such as V600D/E/K and R are found in this group. Class II includes mutants with intermediate to high kinase activation [14••]. While tumor proliferation in high activation class II mutants is more dependent on the MAPK cascade and less on RAS signaling, intermediate activation class II mutants are associated with a lower dependency on the MAPK pathway and higher dependency on RAS signaling [14••]. Class II BRAF mutants tend to have lower kinase activity than class I mutants such as V600E but higher than wild-type BRAF. Class III BRAF mutations are characterized by a low kinase activity or lack thereof altogether [14••]. Clinically, a more aggressive course of disease can often be observed in class II and III BRAF-mutated melanomas than in class I melanomas [14••].

## BRAF V600 mutations (other than V600 E/K)

BRAF V600 mutations belong to class I hotspot mutations resulting in high kinase activity [14••]. For instance, kinase activity in V600E is estimated to be 500–700-fold higher than in wild-type BRAF [19]. Large randomized controlled trials on BRAF inhibition plus MEK inhibition (combi-dv, Columbus, coBRIM) have revealed good overall response of 63 to 68% and improvement of PFS and OS for the most prevalent V600 mutations, V600E and V600K [3••, 9••], [20••]. The clinical behavior of rarer V600 mutations such as V600R, V600D, V600M, V600_K601, V600G, V600L, or V600_S602delinsDT was not assessed [13••]. Prevalence of such rare mutations appears to increase with age and have been found more frequently in male patients with heavy sun exposure [6].

BRAF V600R is the third most common BRAF mutation in malignant melanoma, accounting for approximately 3–7% [21]. It is a class I BRAF mutation with high kinase activity [19]. Retrospective analyses of BRAF V600R-mutated metastatic melanoma propose a response to BRAFi monotherapy in 27 to 83% and to BRAFi plus MEKi combination therapy in 55% of cases with a significant benefit for PFS (3.8 vs. 8.0 months) and OS (7.3 vs. 22.9 months) with the combination therapy when compared to BRAFi monotherapy [13••, 21]. In vitro analysis showed similar functional activity for BRAF V600R and V600K mutations which also resonates with real-world clinical data showing similar response of both mutations to therapeutic MAPK inhibition [19].

There is only limited clinical data available for other rare V600 BRAF mutations such as V600D/G/L/M or V600_K601. As class I mutants, V600-mutated melanomas show high kinase activity and tumor growth heavily relies on the MAPK pathway although its degree varies for different mutants [14••]. Retrospective data of a cohort of 58 patients with metastatic melanoma harboring a BRAF V600 mutation other than V600E/K revealed a response to MAPK inhibition in 45% [13••]. Thus, although less pronounced than in BRAF V600E/K, rare V600 mutants appear to be prone to benefit from management with BRAF inhibitors (BRAFi) plus MEK inhibitors (MEKi).

## Non-V600 mutations

Presence of non-V600 mutations is suspected in 5–16% of melanomas [14••], [22•]. Next-generation sequencing has improved the detection and further characterization of driver mutations in metastatic melanoma. Multiple non-V600 BRAF mutations have been described in anecdotal reports in the literature. Yet, therapeutic efficacy of MAPK inhibition in these mutants remains unclear due to their scarce nature and genetic heterogeneity.

While BRAF V600E mutants achieve monomer activation of the MAPK cascade in the absence of activated RAS (class I mutations), non-V600 BRAF mutants depend on the formation of constitutive dimers for activation (class II) or fail to activate it entirely (class III) [14••]. Shorter survival times for patients with class II or III BRAF mutations compared to class I mutations were described for both lung cancer and advanced melanoma [14••, 18••].

Data from an international retrospective analysis of the response to BRAF/MEK inhibition in 96 advanced melanoma patients with rare BRAF V600 (*n* = 58) and non-V600 mutations (*n* = 38) revealed an overall response in 45% of V600 mutated patients but only 18% of non-V600 patients [13••]. The non-V600 cohort consisted mainly of class II mutations such as L597P/Q/R/S (39%), K601E (29%), G469R/S/A (13%), and several unique mutations (18%) including class III BRAF mutations (A598V, 1596_1597insTAC, T599_V600insT, D594G, and G593D). In these mutations, MEKi monotherapy and BRAFi/MEKi combination also showed a significant advantage for PFS compared to BRAF inhibition alone.

### L597P/Q/R/S

L597Q/R/S are neighboring oncogenic mutations on the BRAF gene with a 138-fold increased kinase activity [23]. L597P has not been functionally or clinically validated but is considered a class II hotspot mutation and likely oncogenic due to its vicinity to L597Q/R/S [14••, 24–26]. Elevated phosphorylated MEK and ERK concentration was revealed for BRAF L597Q/R/S expression, and in vitro and in vivo tumor regression was shown for L597S- and L597Q-mutated melanoma when treated with a MEKi [24, 27]. Two case reports with L597R- and L597S-mutated melanoma confirmed response of this mutation to MEK inhibition [27]. Another case reported response of L597R to BRAFi with vemurafenib [28]. Contradictory findings were reported by Kim et al. with no response to either vemurafenib or trametinib in two L597R-mutated melanoma patients [29]. Dankner et al. generated patient-derived xenografts bearing an L597S mutation which responded to BRAFi, MEKi, and BRAFi plus MEKi combination with best response to the combination. The same group reported response to treatment with BRAFi/MEKi combination in two L597S-mutated melanoma patients [30•]. A retrospective analysis of 15 patients with L597P/Q/R/S mutation showed no response to BRAFi monotherapy, response in one patient with L597Q to MEKi monotherapy, and response in two of nine patients (both L597S) to a BRAFi/MEKi combination treatment, resulting in a response rate of 20% and DCR of 44% [13••]. Although limited by low patient volume, this analysis did not show any significant advantage for either PFS or OS with the combination vs. BRAFi or MEKi monotherapy.

### K601E

K601E is classified as a highly kinase activating type II mutation [14••]. Yet, clinical reports have not shown any response of BRAF K601E-mutated melanoma to either BRAFi or BRAFi/MEKi combination therapy [29, 31]. Further retrospective data of eleven patients showed no response to BRAFi in six patients, no response to MEKi in one patient, and response in only one of four patients treated with a BRAFi/MEKi combination, resulting in an ORR of 9% and DCR of 45% [13••]. However, other reports by Kim et al. reported a response to monotherapy with the MEKi trametinib in one patient, and Bowyer et al. had a response in three of four patients [25, 32].

### G469R/S/A

BRAF G469 is categorized as an intermediate to high activating class II mutation [14••, 17•]. Melanomas harboring this mutation are dependent on RAS-signaling and sensitive to ERK-mediated feedback [17•]. Yet, ERK activation is often less pronounced than in class I and II mutations and may induce insufficient feedback to inhibit RAS. MEK inhibitors may show some efficacy for BRAF mutants in which RAS signaling gains importance [17•]. Retrospective data of five advanced melanoma patients with a BRAF mutation in G469R/S/A/T17OdelinsAK did not show any response to BRAFi monotherapy, but two of three patients responded to a combination therapy with BRAFi plus MEKi (60%).

### Other rare non-V600 mutations

Other rare BRAF non-V600 mutations are so low in prevalence that their occurrence may not even be described in the literature. Thus, it is important to further collect data from next-generation sequencing and connect them with available clinical data from other oncologic centers to conceptualize therapeutic approaches for patients harboring such mutations. Often these mutations are low-kinase activating class II mutations (such as A598V) or lack kinase activity altogether (class III, for instance D594G).

## Targeted therapy for non-V600-mutated melanoma

Clinical efficacy of MAPK inhibition for non-V600 BRAF-mutated melanoma is generally lower than for V600 mutations [13••]. While BRAFi/MEKi monotherapies and combinations have shown high ORR and significantly prolonged PFS and OS in V600E and, to a lesser extent, V600K-mutated melanoma [3••–4••, 9••, 33–34], efficacy in non-V600 mutations depends on their role of BRAF as a genetic driver for tumor proliferation and the existence of other concurrent mutations in key cell signaling pathways [22]. As patients with melanomas harboring less frequent BRAF mutations have been excluded from randomized controlled clinical trials, there is no evidence-based data available on response and survival benefit with BRAFi/MEKi for these patients. The increased use of next-generation sequencing instead of targeted BRAF V600 sequencing and further characterization and categorization of BRAF mutants as class I–III mutations has helped to estimate a possible clinical benefit from available targeted therapies.

ERK signaling requires RAS-induced RAF dimerization and is limited by feedback [35]. Mutants with activated BRAF evade feedback inhibition of RAS by activated monomers (class I) or constitutive RAS-independent dimers (class II) [35]. RAF inhibitors effectively inhibit mutant monomers, but not dimers [35]. The stronger BRAF is activated, the higher a response to a targeted therapy with BRAF inhibitor plus MEK inhibitor can be expected. Hence, the best response to BRAFi/MEKi is seen in V600E-mutated melanomas, followed to a lesser extent by V600K-mutated melanomas in which alternative pathways, such as phosphoinositide (PI) 3-kinas (PIK3-AKT), may gain more importance for tumor proliferation [34].

The most frequent non-V600 BRAF mutations are L597P/Q/R/S (15%) and K601E (11%), all of which are class II BRAF mutations [27]. In intermediate activating mutants such as the V600-neighboring mutants K601E, L597, and G469A, suboptimal catalytic kinase efficiency due to different molecular structures has been shown [19]. In these mutations, the activation of other tumor drivers such as wild-type C-RAF and subsequent elevation of ERK activity appear to play an important role in pathogenesis. Reports linking elevated C-RAF protein levels to drug resistance to BRAF inhibition support this hypothesis [36].

While class I monomer signaling is disengaged from receptor tyrosine kinase (RTK) and RAS signaling, class II mutants activating MEK/ERK as dimers show some RTK and RAS signaling and require MEK inhibition (trametinib, cobimetinib, binimetinib), pan-RAF inhibitors (sorafenib), or paradox-breaking BRAF dimerization inhibitors [14••].

## MEK inhibition

In BRAF- and NRAS-mutated melanoma, tumor proliferation and cell survival are substantially driven by increased activation of the MAPK pathway [37]. Through the RAS-RAF-MEK-ERK cascade, proliferative signals are transmitted into the nucleus promoting tumor growth. MEK inhibitors approved (in combination with a BRAF inhibitor) for therapy of metastatic melanoma with a BRAF V600E or V600K mutation are trametinib (in combination with dabrafenib), cobimetinib (in combination with vemurafenib), and binimetinib (in combination with encorafenib) [3••–4••, 9••]. Due to a lack of specific NRAS-targeted therapeutics, MEK inhibitors are often used in NRAS-mutated melanomas which have failed therapy with immune checkpoint inhibitors [38••]. While MEK inhibitors may fully block tumor proliferation in BRAF-dependent melanomas, RAS mutants are only partially inhibited [39]. Hence, further knowledge about dependency on the MAPK pathway and NRAS feedback in unique BRAF mutants is essential to predict therapeutic benefit for targeted therapy.

### Trametinib

The first MEK inhibitor approved for advanced melanoma by the FDA was trametinib in 2013, followed by cobimetinib in 2015 and binimetinib in 2018. Trametinib is a selective MEK1/2 kinase inhibitor indicated, in combination with the BRAF inhibitor dabrafenib, for the treatment of patients with unresectable or metastatic melanoma with BRAF V600E or V600K mutations or in the adjuvant setting of V600E- or V600K-positive melanomas. It is also the only MEK inhibitor monotherapy approved by the US Food and Drug Administration [22•].

The recommended dosage is 2 mg orally every 24 h at least 1 h before or 2 h after a meal.

Data from the phase 3 METRIC study comparing trametinib vs. chemotherapy in V600E/K mutated melanoma revealed clear superiority of MEK inhibition with an ORR of 24% for trametinib vs. 7% for chemotherapy and median PFS of 4.8 months vs. 1.5 months, respectively [40••].

In vitro and clinical data suggests some efficacy of MEK inhibitors for non-V600 mutations [24]. Nebhan et al. documented a case series of nine patients with non-V600 BRAF-mutated advanced melanoma treated with trametinib [22]. A response was reported for 67% of patients with high kinase-activating and 17% of patients with low kinase-activating non-V600 mutations. Three patients who were classified as class III BRAF mutants did not respond to trametinib at all.

Data from a large case collection of melanoma patients with rare BRAF mutants showed in a subpopulation of 38 non-V600 BRAF-mutated patients an ORR of 18% for MAPK inhibition with none of 15 patients responding to BRAFi, two of five patients (40%) responding to MEKi, and five of 18 patients (28%) responding to a BRAFi/MEKi combination [13••]. The lack of response to BRAFi monotherapy supports the hypothesis that MEK inhibition may be responsible for any therapeutic effect seen with MAPK inhibition in non-V600 BRAF mutations. A subanalysis of the most frequent non-V600 mutations, L597P/Q/R/S, K601E, and G469R/S/A (81.6% of non-V600 mutations in this study), all of which are high kinase activating class II BRAF mutations, showed an ORR of 18% with none of 13 patients responding to BRAFi monotherapy, one of two patients responding to MEKi monotherapy, and five of 16 patients (31%) responding to BRAFi/MEKi combination.

Thus, depending on the class of kinase activation, MEKi currently remains an important therapeutic option for metastatic melanoma patients with an activating non-V600 BRAF mutation who have failed or are not eligible for immune checkpoint inhibitors in the first line.

## NRAS-mutated melanoma

After BRAF, RAS is the second most frequent driver mutation in cutaneous melanoma [2]. NRAS mutations are generally mutually exclusive with BRAF V600E/K mutations [41]. Hotspot mutations in the NRAS gene, especially codon Q61, less frequently G12 and G13, can be found in approximately 10–25% of melanomas [2]. NRAS mutation-driven melanomas are more frequently seen in primary tumors developing as nodular melanomas on sun-exposed skin [42].

In vitro investigations suggested sensitivity of NRAS-mutated melanomas to MEK inhibition [38••]. A phase II study showed a response of MEKi treatment in 15% of NRAS-mutated metastatic melanoma [43]. In a phase III study (NEMO) with 402 patients with NRAS-mutated melanoma assigned to either a group receiving binimetinib (*n* = 269) or dacarbazine (*n* = 133), overall response rate (ORR) was 15% vs. 7%, progression-free survival (PFS) 2.8 vs. 1.5 months, and median overall survival (OS) 11 months vs. 10.1 months, respectively [38]. A significant benefit could only be shown for PFS, not OS.

New therapeutic approaches in NRAS mutants may comprise combination of MEK inhibitors with the CDK4/6 inhibitor ribociclib or MDM2 antagonists [44].

## C-Kit-mutated melanoma

C-Kit gene mutations are especially found in acral and mucosal melanomas [45]. While less than 5% of melanomas in Caucasians harbor a c-kit mutation, it is the most prevalent mutation in Asians accounting for over 70% in the Chinese population [46]. Especially in patients with melanomas harboring a c-Kit mutation, a therapeutic approach with the multi-kinase inhibitor imatinib may be considered [45]. Clinical efficacy of imatinib is associated with hotspot mutations in exon 11, 13, and 17 which were found in 60% of a Chinese cohort of 78 patients with advanced c-kit-mutated melanoma [46]. ORR and disease control rate (DCR) were 21.8% and 60.3%, respectively. Thus, imatinib maintains an important role as a second-line targeted therapy for patients with advanced melanoma harboring a c-kit mutation.

### Imatinib

Imatinib (Gleevec) is a multikinase inhibitor of bcr-abl, c-KIT, and PDGF-R indicated for bcr-abl-positive chronic myeloid leukemia and dermatofibrosarcoma protuberans. Melanomas with a mutation in c-kit may benefit from a targeted therapy with imatinib as “off-label” use. Approximately 1% of melanomas, especially acral and mucosal melanomas, harbor a c-Kit mutation.

The recommended dosage of imatinib is 400 mg orally twice daily taken with a meal and a large glass of water. Imatinib should not be administered to breastfeeding patients. Contraception should be granted in male and female patients of reproductive potential.

CYP3A4 inducers may decrease the systemic concentration of imatinib, while CYP3A4 inhibitors may increase imatinib concentration. As imatinib is a CYP3A4 inhibitor itself, caution is advised with concomitant medications which may require dose modifications.

The most common adverse reactions (≥10%) are edema, nausea, vomiting, muscle cramps, musculoskeletal pain, diarrhea, rash, fatigue, and abdominal pain [47].

## Emerging therapies

Due to the relative rarity and great variety of non-V600 BRAF mutations in melanoma, development of a randomized controlled trial for targeted therapies remains challenging. However, some approaches have involved the combination of MEK inhibition with BRAF or other receptor-tyrosine kinase inhibitors or single-agent ERK inhibition [17•, 48].

The selective adenosine triphosphate (ATP) competitive inhibitor LXH254 is a novel RAF inhibitor, which has shown ability to inhibit monomeric BRAF (class I mutations), as well as dimerized (class II) BRAF and CRAF, while sparing ARAF [49]. LXH254 may thereby help overcome intrinsic and acquired resistance to BRAF-targeted therapy in BRAFV600-mutated melanomas and be a novel therapeutic approach for NRAS-mutated patients.

Other possible therapeutic novelties that may (also in combination) be beneficial as targeted therapies for non-V600-mutated melanoma patients include ribociclib (cyclin D1/CDK4 and CDK 6 inhibitor) or LTT462 (ERK1/2 kinase inhibitor) [44].

## Conclusion

Non-V600 BRAF-mutated melanoma remains a therapeutic challenge if treatment with checkpoint inhibitors fails. BRAF and/or MEK inhibition has shown some efficacy in unique mutations, yet, long-term tumor control as seen in a third of V600E/K mutations is uncommon. The categorization into different catalytic activity classes may help to predict a possible benefit for patients harboring non-V600 mutations. Further research and clinical data are warranted to better understand efficacy of MAPK inhibition in unique mutants. Next-generation sequencing may identify other mutations relevant as therapeutic targets for the treatment of advanced melanoma.
